# IL‐22Rα1 restrains pancreatic injury independently of its C‐terminal STAT3‐amplifying domain

**DOI:** 10.1002/ccs3.70098

**Published:** 2026-07-28

**Authors:** Léna Puigdevall, Clara Stewardson, Lysa Dhulst, Emilie Hendrickx, Laure Dumoutier

**Affiliations:** ^1^ Experimental Medicine Unit de Duve Institute UCLouvain Brussels Belgium

**Keywords:** acute pancreatitis, biology, cancer research, cell biology, cytokine, inflammation, pancreatitis, phenotype, regulator, stat3

## Abstract

Interleukin‐22 (IL‐22) is a cytokine that regulates tissue inflammation and repair, primarily through activation of STAT3 signaling. Although its protective effects in acute pancreatitis (AP) are well established, the specific contribution of its receptor, IL‐22Ra1, and the underlying signaling mechanisms remain incompletely defined. Notably, the C‐terminal region of IL‐22Ra1, although devoid of tyrosine residues, has been reported to promote STAT3 activation through unconventional mechanisms. Here, we investigated the role of IL‐22Ra1 and its C‐terminal (STAT3‐associated) domain in a mouse model of caerulein‐induced AP. We show that deletion of the C‐terminal region of IL‐22Ra1 markedly reduces STAT3 activation in the pancreas, confirming its role in amplifying canonical signaling. Unexpectedly, this reduction does not worsen pancreatic injury. In contrast, both IL‐22‐ and IL‐22Ra1‐deficient mice develop more severe pancreatitis, characterized by increased serum amylase levels, exacerbated inflammatory responses, and enhanced tissue damage. Notably, IL‐22Ra1 deficiency results in a more severe phenotype than IL‐22 deficiency alone, indicating that IL‐22Ra1 mediates protective effects beyond IL‐22 signaling. Consistently, acinar‐to‐ductal metaplasia during the regenerative phase is impaired in both IL‐22‐ and IL‐22Ra1‐deficient mice but preserved upon C‐terminal truncation. Together, these findings identify IL‐22Ra1 as a central regulator of pancreatic protection through mechanisms that are independent of maximal STAT3 activation and extend beyond IL‐22 itself, suggesting the involvement of additional IL‐22Ra1 ligands. These results support the exploration of receptor‐activating strategies, such as agonistic antibodies, to promote tissue protection and regeneration.

## Graphical abstract

IL‐22R, and to a lesser extent IL‐22, are key drivers of protection in acute pancreatitis. Although the C‐terminal domain of IL‐22R enhances STAT3 activation in the pancreas, it is not required for disease protection. Classical IL‐22R signaling alone is sufficient to control inflammation and promote pancreatic recovery.

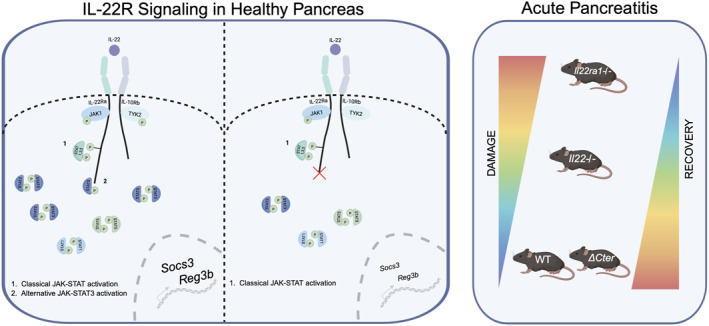

## 1 INTRODUCTION

Pancreatitis, an inflammatory disorder of the pancreas, represents a complex and multifactorial disease characterized by local tissue injury and systemic inflammatory responses. Although initial inciting events—such as gallstones, alcohol abuse, or metabolic disturbances—trigger pancreatic acinar cell injury, the subsequent inflammatory cascade largely determines disease severity and progression. Chronic inflammation can further lead to genetic damage and uncontrolled cell proliferation, thereby increasing the risk of pancreatic cancer. Central to this inflammatory cascade is the dysregulated release of cytokines. Among them, IL‐22 has emerged as a key protective mediator in acute pancreatitis (AP). The strongest clinical evidence supporting a role for IL‐22 in acute pancreatitis is the observation that circulating IL‐22 levels are increased in patients with acute pancreatitis, with its concentration correlating with disease severity.^1^ Exogenous IL‐22 administration in caerulein‐induced pancreatitis markedly reduces serum amylase, mitigates histopathological damage, and restrains excessive autophagy.^2^

Interleukin‐22 (IL‐22) is a member of the IL‐10 family of cytokines^3^ produced by both innate and adaptive immune cells. Depending on the context, IL‐22 can have beneficial or harmful effects.^4^ Under controlled conditions, IL‐22 promotes tissue repair and defense against pathogens in various organs including the skin, gut, and pancreas. However excessive IL‐22 production can contribute to inflammatory and neoplastic disorders such as psoriasis and cancer.^5^ IL‐22 signals through a heterodimeric receptor composed of IL‐22Rα, which is selectively expressed on epithelial cells, and the ubiquitously expressed IL‐10Rβ chain. Upon ligand binding, the receptor primarily activates the JAK‐STAT (Janus kinase‐signal transducer and activator of transcription) signaling pathway. Of note, the IL‐22Rα chain can also pair with the IL‐20Rβ chain, allowing the binding of other cytokines of the same family, namely IL‐20 and IL‐24.^6^

In the canonical JAK‐STAT pathway, receptor engagement leads to activation of JAK1, which phosphorylates tyrosine residues within the cytoplasmic tail of the receptor. These phosphorylated tyrosine residues serve as docking sites for STAT proteins. Among them, STAT3 is the primary STAT activated by IL‐22.^7^ Once recruited and phosphorylated, STATs dimerize and migrate to the nucleus to regulate the expression of target genes.^8^ Although this classical JAK‐STAT pathway relies on the receptor's tyrosine phosphorylation, alternative phosphotyrosine‐independent signaling have been described for several receptors.^8^, ^9^, ^10^, ^11^, ^12^

Accordingly, our previous works demonstrated that IL‐22R's tyrosines are not absolutely required for STAT3 activation. In vitro, mutation of all receptor's tyrosines to phenylalanine residues did not abolish STAT3 activation although STAT1 and 5 activation was impaired. We further showed that STAT3 is preassociated with the C‐terminal (C‐ter) part of the receptor, a tyrosine‐free domain, allowing its direct phosphorylation by JAKs independently of receptor's tyrosine phosphorylation.^7^ Therefore, canonical and alternative signaling pathways cooperate to induce a massive activation of STAT3 upon IL‐22 stimulation.

We also demonstrated that the C‐ter part of IL22 Rα plays a major role in IL‐22 signaling in the skin, but only a minor one in the gut. In a psoriasis‐like dermatitis model triggered by Imiquimod, where IL‐22 and STAT3 exert pathogenic effects, expression of a C‐terminally truncated IL‐22Rα (Δ*Cter* ^*mut/mut*^ mice) dampens skin lesions, phenocopying *Il22ra1−/−* mice.^13^ In contrast, in experimental colitis model, where IL‐22 is protective, C‐ter truncation has no significant effect. These findings suggest that the role of the alternative signaling pathway is context‐dependent and may be particularly relevant in pathologies associated with massive STAT3 activation. Targeting this pathway could therefore allow to partially reduce the deleterious effects of IL‐22 in psoriasis while preserving its beneficial functions in tissue repair and barrier defense at other sites.^14^, ^15^, ^16^, ^17^

Given the high expression of IL22RA1 in the pancreas, we next focused on pancreatic disease. Although exogenous IL‐22 administration has been consistently reported to confer protection during pancreatitis,^2^, ^18^, ^19^ the consequence of IL‐22 or IL‐22 receptor deficiency remains largely unresolved. This includes their role on acinar‐to‐ductal metaplasia, a critical process in pancreatic injury and repair.

Mechanistically, the IL‐22‐STAT3‐pancreatitis associated protein (PAP1) axis has emerged as a central pathway in pancreatic protection.^2^, ^20^ However, the specific contribution of the C‐terminal domain of the IL‐22 receptor—known to mediate potent STAT3 activation—has not been investigated in the context of caerulein‐induced pancreatitis. Addressing this gap may uncover previously unrecognized regulatory mechanisms governing pancreatic resilience and regeneration.

Here, we identify IL‐22Ra1 as a key player in pancreatic inflammation, exerting a more prominent protective role than the cytokine itself. Although the C‐terminal region of IL‐22Rα1 contributes to STAT3 activation in the pancreas, its absence—despite reducing STAT3 signaling—does not exacerbate caerulein‐induced pancreatitis.

## 2 METHODS

### 2.1 Mice and reagents

All mice used in this study were bred in our animal facility under specific pathogen‐free conditions. *Il22* ^*−/−*^ and *Il22ra1* ^*−/−*^ mice were generated in 129sv/C57BL/6 background in our laboratory as previously described.^21^, ^22^ Mice were backcrossed 20 times on C57BL/6 and kept in a littermate background. *Il22* ^*+/+*^ or *Il22ra1* ^*+/+*^ mice served as control. To generate Δ*Cter* ^*mut/mut*^ mice, we used a cloning‐free CRISPR/Cas9 system to target the mouse *Il22ra1* gene.^23^ Mice carrying the mutation were crossed with C57BL/6 female, and the strain was kept in a littermate background (B6D2x C57BL/6). Mice were backcrossed 10 times on C57BL/6. Δ*Cter* ^*wt/wt*^ mice were used as a control of Δ*Cter* ^*mut/mut*^ mice. Mice were selected based on the presence of the mutation in the *Il22ra1* by PCR genotyping. The obtention of these mice was previously described.^13^

All mouse experiments were conducted in accordance with national and institutional guidelines and were approved by the local ethical committee (LA 1230653‐2023/UCL/MD/08).

Recombinant mouse IL‐22 was produced in *E*. *coli* as described previously.^24^

### 2.2 Mice genotyping

Genomic DNA was isolated from tail biopsies collected from littermate mice. Genotyping was performed by polymerase chain reaction (PCR) using allele‐specific primer sets (Table 1) designed to detect either the wild‐type (WT) or knockout (KO) allele. For each DNA sample, two independent PCR reactions were carried out: one using WT‐specific primers and another using KO‐specific primers. PCR products were separated by agarose gel electrophoresis. Mice were classified as wild‐type when amplification was detected only in the WT‐specific PCR, knockout when amplification was detected only in the KO‐specific PCR, and heterozygous when amplification was observed in both reactions. Genotyping data of all 3 mouse strains are displayed in Supporting Information S1 Figure S1.

**TABLE 1:** Genotyping primers.

Mouse genotype	Forward primer (5′‐3′)	Reverse primer (5′‐3′)
*Il22−/−*	AGCGCATGCTCCAGACTGCC	AATCTATGAAGTTGGTGGGA
*Il22+/+*	GCTTACCTGTTTAGGTGTCTCTG	AATCTATGAAGTTGGTGGGA
*Il22ra1−/−*	GGATGTGCTGCAAGGCGATTAAG	GGACAGACGATATGCAACACAC
*Il22ra1+/+*	GTCTCCTTCAACACGTGAAA	GAAACGATCAGTCATCTTTGTG
*ΔCter+/+*	GCTGCCCTGCTTCTTATGCTGT	GCCCTCGATCTGCACAGAGG
*ΔCter−/−*	GCTGCCCTGCTTCTTATGCTGT	GCCCTCGATCTGCACAGATT

### 2.3 Caerulein‐induced pancreatitis model

Acute pancreatitis was induced by six hourly intraperitoneal injections of caerulein (Eurogentec, 75 μg/kg). Blood samples were collected 20 hours (referred to as Day 1, D1) or 3 days (D3) after the last injection. Mice were sacrificed either at D1 or D3 following caerulein/PBS injection therefore corresponding to independent experimental cohorts rather than a longitudinal follow‐up. When the mice were sacrificed, their pancreas was harvested, weighted and sectioned for RNA extractions and histological staining.

### 2.4 Blood chemistry

Levels of serum amylases were assessed using FUJI DRI‐CHEM Slides (Fujifilm, LIP‐16654114, AMYL‐15827934) read on FUJI DRI‐CHEM NX500 Analyzer.

### 2.5 Western Blot

**FIGURE 1:**
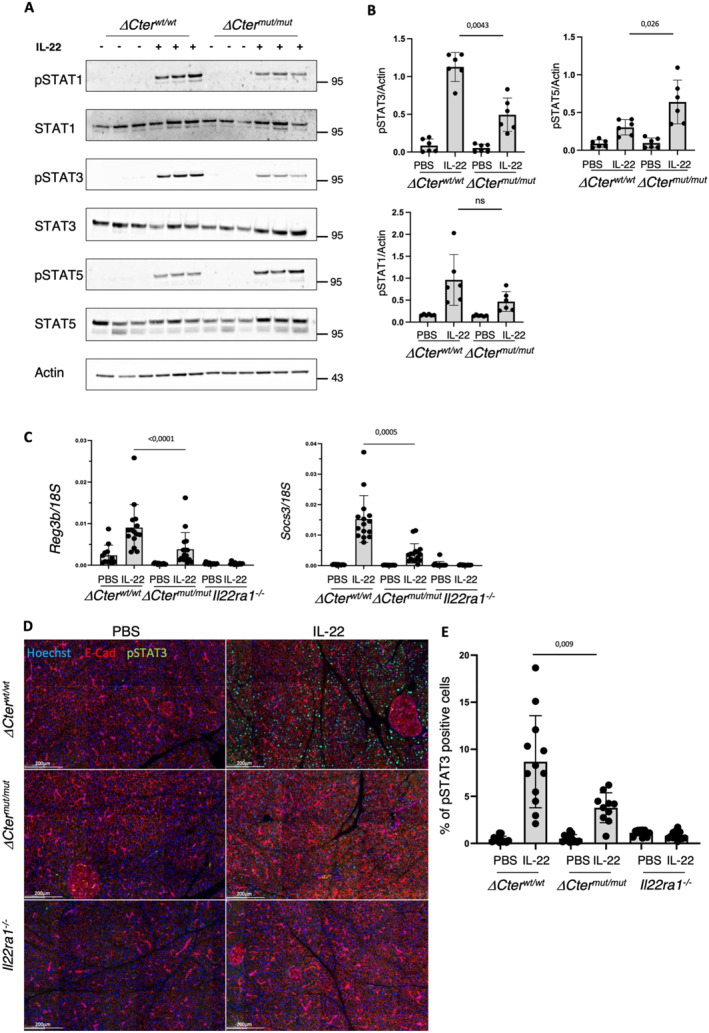
The C‐ter of IL‐22Ra is involved in IL‐22 signaling in the pancreas. (A) Western Blot analysis of IL‐22 signaling in the pancreas. 30 minutes after PBS or IL‐22 injection (10 μg/mouse), pancreas pieces were cut and frozen in liquid N2. Proteins were extracted and charged on gels. Actin is used as loading control. Representative of 2 independent experiments (*n* = 3 mice per group). (B) Quantification of western blots (*n* = 6). (C) qPCR analysis on pancreas from mice injected with PBS or IL‐22 (10 μg/mouse). Mice were dissected 30 minutes after injection. RNA was extracted and gene expression of *Socs3* and *Reg3b* was normalized on *18s* housekeeping gene. Representative of 4 different experiment (*n* = 3 mice per group). Data are shown as mean ± SEM. (D) Immunofluorescent staining on paraffin‐embedded sections of pancreas. 30 minutes after PBS or IL‐22 injection, pancreas pieces were cut and fixed in PFA (4%). 4 μm sections were stained with pSTAT3 (green), E‐cadherin (red), and Hoechst (blue). Representative of 2 independent experiments and 2 different sections per condition (*n* = 3 mice per group). Magnification x20, scale bar = 200 μm. (E) Quantification of pSTAT3 positive cells compared to total acinar cells using the Halo software. Quantifications were performed on two tissue sections per mouse. Statistical analysis: Mann–Whitney (ns = not significant).

Cells were stimulated with control medium or murine IL‐22 (500 ng/mL) for the indicated time points at 37°C. For in vivo experiments, IL‐22 (10 μg/mouse) was injected intraperitoneally, and after 30 minutes, organs were harvested, snap‐frozen in liquid nitrogen, and homogenized using TissueLyser LT (Qiagen, Hilden, Germany). Lysis was performed as previously described.^25^ After protein quantification with Pierce BCA Protein Assay Kit (Thermo Fisher Scientific), a total of 2 or 10 μg of proteins diluted in LDS sample buffer and Sample Reducing Agent (Thermo Fisher Scientific) were loaded on Bolt 4–12% Bis‐tris gels (Thermo Fisher Scientific) and transferred to a nitrocellulose membrane (iBlot Gel Transfer Stacks, Thermo Fisher Scientific). The JAK‐STAT pathway was analyzed using the following antibodies from Cell Signaling Technology: antipY701 STAT1 (#9167), anti‐STAT1 (#9172), anti‐pY705 STAT3 (#9131), antiSTAT3 (#9131), antipY694 STAT5 (#9351), antiSTAT5 (#9363) and antiβ actin (A5441, Sigma). Signal quantification was performed using the Bio1D software. To maintain the readability of the figure and avoid overcrowding, we have not included all *β*‐actin blots in Figure 1. However, all loading controls were used for the densitometric quantification, and each target protein was normalized to its corresponding *β*‐actin before statistical analysis.

### 2.6 RT‐qPCR

IL‐22 (10 μg), diluted in PBS, was administered to mice by intraperitoneal injection, and different organs were collected 1 hour post‐injection. Tissues were incubated in RNAlater (Thermo Fisher Scientific) prior to RNA extraction. Total RNA was isolated from organs using TriPure Isolation Reagent (Roche, Basel, Switzerland) after tissue disruption with TissueLyser LT (Qiagen). The same procedure was applied to extract RNA from organs collected in pancreatitis or tumor models. For in vitro experiments, total RNA was extracted from cells 1 hour after stimulation with control medium or murine IL‐22 (500 ng/mL) using the same TriPure Isolation Reagent. RT‐qPCR was performed on 1 μg of total RNA using oligo(dT) primer (Eurogentec) and Moloney murine leukemia virus reverse transcriptase (Invitrogen). Quantitative PCR (qPCR) amplifications were performed on cDNA corresponding to 25 ng of total RNA, using primer sets and TaqMan probes with qPCR Mastermix TaqMan or SYBR Green (Eurogentec). The sequences of primers and probes are provided in Table 2. Samples were first heated for 10 min at 95°C, before amplification as follows: 40 cycles of two‐step PCR program at 95°C for 10 sec and 60°C for 1 min. For SYBR Green qPCR, a melting curve analysis was carried out by heating the amplicon from 60 to 95°C. A standard curve generated from serial dilution of a known concentrations of a cloned cDNA fragment was used for each gene.

**TABLE 2:** RT‐qPCR primers and probe sequence.

Gene	Forward primer (5′‐3′)	Reverse primer (5′‐3′)	Probe (5′‐3′)
*18s*	GTAACCCGTTGAACCCCATT	CCATCCAATCGGTAGTAGCG	/
*Tnfa*	CATCTTCTCAAAATTCGAGTGACAA	TGGGAGTAGACAAGGTACAACCC	CACGTCGTAGCAAACCACCAAGTGGA
*Socs3*	GCCTCGGGGACCATAGGA	GCATCCCGGGGAGCTAGT	/
*Reg3b*	CTACTGCCTTAGACCGTGCTTTC	GAGTCTTCACATTTTGTCCCTTGTC	GTGAAGTTGCCCTATGTCTGC
*Il6*	CAGAGTCCTTCAGAGAGATACAGAAA	TCCAGCTTATCTGTTAGGAGAGCATT	/
*Il1b*	GACGGACCCCAAAAGATGAAG	CTCTTGTTGATGTGCTGCTGTG	/
*Bcl2l1*	AGGCAGGCGATGAGTTTGAAC	GAACCACACCAGCCACAGTCA	/
*Cxcl3*	CCATCCAGAGCTTGACGGTGAC	TGGACTTGCCGCTCTTCAGTATC	

### 2.7 Histology

Pancreatic tissues were paraffin‐embedded using standard procedure. Sections (4 μm thick) were stained with H&E and scanned using Panoramic 250 (3DHISTECH, Budapest, Hungary). Edema and acinar‐to‐ductal metaplasia were quantified using the Halo software (Indica Labs), based on one section per organ.

Immunofluorescent staining for pSTAT3 and CK19 was performed on paraffin‐embedded tissue sections after antigen retrieval using either citrate buffer or proteinase K, respectively. STAT3 activation was assessed using an anti‐pY705 STAT3 antibody (#9145, dilution of 1/200, Cell Signaling Technology, Danvers, MA). An Anti‒E‐cadherin antibody was used as an epithelial marker (#610181, dilution of 1/250, BD biosciences). CK‐19 antibody was used as an acinar‐to‐ductal metaplasia marker (Merck MABT913 dilution of 1/30 000). Secondary antibodies AF488 Donkey antiRabbit IgG (A21206, Thermo Fisher Scientific), AF488 donkey anti‐rat IgG (A21208, Thermo Fisher Scientific) and AF594 Donkey antiRabbit IgG (A21207, Thermo Fisher Scientific) as well as Hoechst (Sigma‐Aldrich, St. Louis, MO) were applied at 1/1000. Slides were scanned using a Pannoramic 250 scanner (3DHISTECH).

### 2.8 RNA‐scope

RNAscope staining was performed on 4 μm paraffin‐embedded sections of healthy pancreas after 24 hours of fixation in 4% paraformaldehyde (PFA). The RNAscope™Multiplex Fluorescent Reagent Kit v2 (323136, Bio‐Techne‐ ACD bio) was used in combination with a probe mix targeting murine Il22ra1 mRNAs (429971‐C2, Bio‐Techne‐ACD bio) and TSA vivid fluorophore 570 (323272, Bio‐Techne‐ ACD bio). All procedures were carried out according to the manufacturer's instructions (document UM 323100, ACD bio), including the recommended pretreatment conditions for pancreatic tissue. Negative and positive control probes were supplied by the firm. RNAscope staining was followed by an immunohistofluorescent staining of E‐cadherin and DAPI as described above. Sections were mounted using ProLong Gold antifade reagent (P10144, Invitrogen) and scanned with Pannoramic 250 scanner (3DHISTECH).

### 2.9 Statistics

Results are presented as mean ± SEM. Statistical analyses were performed using Prism software (GraphPad Software, La Jolla, CA). As the primary objective of this study was to investigate the impact of the different genotypes, and more specifically the contribution of enhanced STAT3 phosphorylation mediated by the C‐terminal region of IL‐22R, to the severity of caerulein‐induced acute pancreatitis. Accordingly, our major comparisons were between WT and mutant mice subjected to caerulein treatment at the corresponding time points, as these comparisons directly address our central biological hypothesis regarding genotype‐dependent susceptibility to pancreatic injury.

Statistical significance between c aerulein‐treated groups of WT, *Il22* ^*−/−*^, *Il22ra1* ^*−/−*^ or Δ*Cter* ^*mut/mut*^ mice was assessed with the Mann–Whitney test (nonparametric conditions). For comparisons involving more than two groups, statistical significance was assessed using the Kruskal‒Wallis test.

## 3 RESULTS

### 3.1 The C‐ter region of IL‐22Ra is required for optimal IL‐22‐dependent STAT3 response in the pancreas

To investigate the effect of the alternative STAT3 activation pathway in vivo in the pancreas, we used our Δ*Cter* ^*mut/mut*^ mice lacking the C‐terminal part of the IL‐22Rα. These mice were generated using the CRISPR/Cas9 approach. A STOP codon was inserted at position 500 of *Il22ra1* gene, nine residues downstream of the last tyrosine (Tyr491), generating a truncated receptor. ΔCter‐mutant mice allowed to study the effect of the alternative, STAT3‐independent, pathway in different organs and were always compared to Δ*Cter* ^*wt/wt*^ littermate mice, harboring the full‐length receptor. IL‐22 responsiveness in the pancreas was analyzed after intraperitoneal (i.p) injection of the cytokine. Western blot analysis showed a marked reduction in STAT3 phosphorylation (pSTAT3) in the pancreas of Δ*Cter* ^*mut/mut*^ mice compared with control littermate Δ*Cter* ^*wt/wt*^ mice (Figure 1A and quantification in 1B). In contrast, STAT1 phosphorylation remains unchanged, whereas STAT5 phosphorylation was increased, possibly reflecting a compensatory mechanism. This increase has previously been reported in the skin of Δ*Cter* ^*mut/mut*^ mice after IL‐22 stimulation.^13^ Consistent with these findings, RT‐qPCR showed lower expression of the STAT3 target genes *Reg3b* and *Socs3* (Figure 1C), in the pancreas of Δ*Cter* ^*mut/mut*^ mice compared to control mice.

To identify IL‐22 responsive cells within the pancreas, immunofluorescent staining for pSTAT3 was performed (Figure 1D). pSTAT3 staining was mainly detected in acinar cells and not in islet cells despite previous reports describing IL‐22Rα expression in both alpha and beta cells of the Langerhans islets.^26^ This expression pattern was further validated by RNAscope staining, that confirmed *Il22ra1* mRNA expression in the pancreas (Supporting Information S1 Figure S2). Quantification of pSTAT3‐positive cells across different pancreatic sections using the Halo software (Figure 1E) demonstrated a significant reduction in Δ*Cter* ^*mut/mut*^ mice compared with controls. Altogether, these data suggest that the C‐ter part of IL‐22Rα plays an important role in IL‐22 signaling in the pancreas, particularly through STAT3 activation. Consequently, this region may therefore contribute to IL‐22‐driven pancreatic inflammation.

### 3.2 Deletion of IL‐22 or IL‐22Ra, but not the C‐terminal region, worsen the inflammatory phase of acute pancreatitis

**FIGURE 2:**
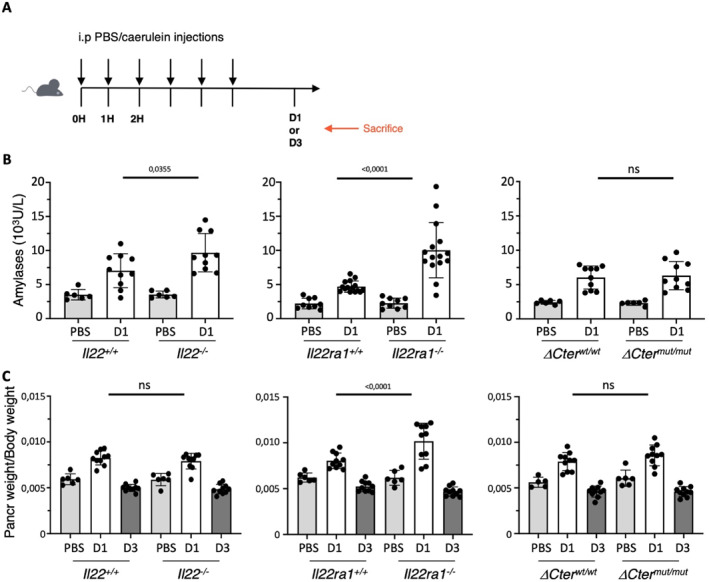
Amylase levels and pancreas weight as indicators of the severity of pancreatitis in *Il‐22*, *Il22ra1* and Δ*Cter* mice. (A) Schematic illustration of caerulein‐induced pancreatitis model: mice were injected intraperitoneally with a high dose of caerulein (75 μg/kg in PBS) every hour for 6 hours and sacrificed 1 day (D1) or 3 days (D3) after initial caerulein injection. Mice were sacrificed either at D1 or D3 following caerulein/PBS injection therefore corresponding to independent experimental cohorts. (B) Amylase levels (U/L) measured on serum of mice with FUJI DRI‐CHEM slides read on FUJI DRI‐CHEM NX500 Analyzer. (C) Pancreas weight to total body weight ratio analysis. Data are shown as mean ± SEM and are a pool of 2 (or 3 for *Il22ra* mice) independent experiments with 3–5 mice/group. Statistical analysis between WT and mutant mice treated with caerulein at the same time‐point: Mann–Whitney (ns = not significant).

The role of IL22 Rα and its alternative signaling pathway was investigated in the pancreas using the well‐established caerulein‐induced model of acute pancreatitis. Repeated intraperitoneal injections of caerulein, a cholecystokinin analog, cause acute pancreatitis (AP) in mice. Following 6 hourly injections, the mice were bled and sacrificed either 1 day or 3 days after the first injection (Figure 2A). The D1 (1 day) time point, corresponds to the initial damage phase of AP, characterized by edema and inflammation, whereas the “3‐day” (3D) time point reflects the regenerative phase, during which pancreatic cells display a ductal phenotype via a protective process known as acinar‐to‐ductal metaplasia (ADM).

At day one, serum amylase levels were significantly increased in *Il22* ^*−/−*^ and *Il22ra1* ^*−/−*^ mice compared with their respective control littermate mice, indicating a more severe form of pancreatitis in these mice. In contrast, deletion of the C‐terminal region (Δ*Cter* ^*mut/mut*^ mice) did not result in elevate amylase levels compared with Δ*Cter* ^*wt/wt*^ littermate mice (Figure 2B). We also assessed the ratio of pancreatic weight to total body weight, a parameter that generally increases during the acute phase due to pancreatic edema and inflammation. A significant increase in this ratio at one day post caerulein injection was observed in *Il22ra1* ^*−/−*^ mice but not in *Il22* ^*−/−*^ and Δ*Cter* ^*mut/mut*^ mice (Figure 2C).

**FIGURE 3:**
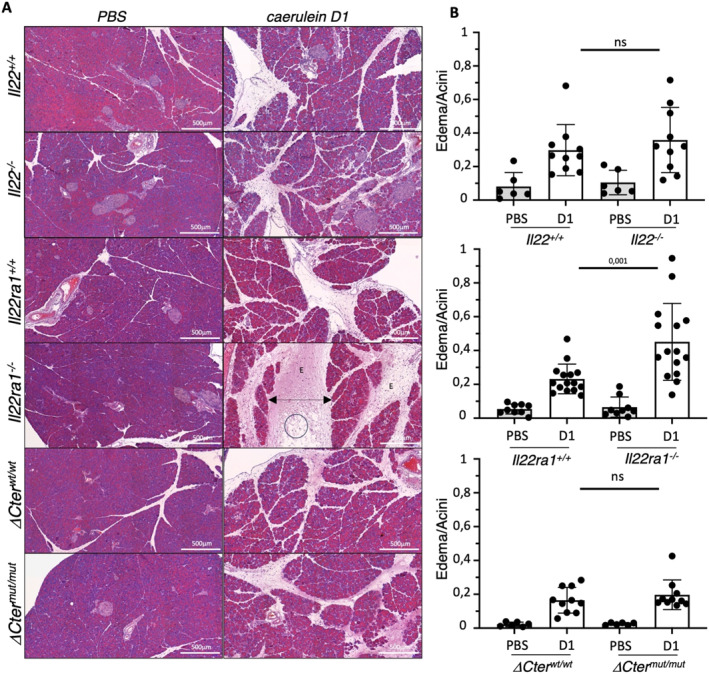
Assessment of the inflammation induced after acute pancreatitis in *Il22* ^*−/−*^, *Il22ra1* ^*−/−*^ and Δ*Cter* ^*mut/mut*^ mice. (A) H&E staining of pancreas sections of mice treated with caerulein or PBS at day 1 (D1) after initiation of pancreatitis. Magnification x10, scale bar = 500 μm. One representative picture is shown for each group (*n* = 3–5 mice/group). E = presence of edema, double arrow represents the septa enlargement and circled are infiltrating leucocytes. Quantification of edema‐to‐acini ratio in indicated genotypes (B) was performed using the Halo software. D1 = 1 day after caerulein injection. Data are shown as mean ± SEM and are a pool of 2 (or 3 for *Il22ra* mice) independent experiments with 3–5 mice/group. Statistical analysis between WT and mutant mice treated with caerulein at the same time‐point: Mann–Whitney (ns = not significant).

To further evaluate edema and inflammation during AP, sections of pancreas were stained with Hemalun Eosine (H&E) (Figure 3A,B), and the expression of inflammatory markers was evaluated by qPCR (Figure 3C). Histological analysis of H&E staining shows that, at one day, pancreatic tissue exhibited typical features of acute injuries, including edema, enlarged septa, necrosis and immune cell infiltration (Figure 3A). Quantification of edema‐to‐acini ratio using the Halo software showed increased tissue damage in *Il22ra1* ^*−/−*^ mice compared with wild‐type counterparts, whereas no significant differences were observed in *Il22* ^*−/−*^ mice or Δ*Cter* ^*mut/mut*^ mice relative to their respective littermate controls (Figure 3B).

**FIGURE 4:**
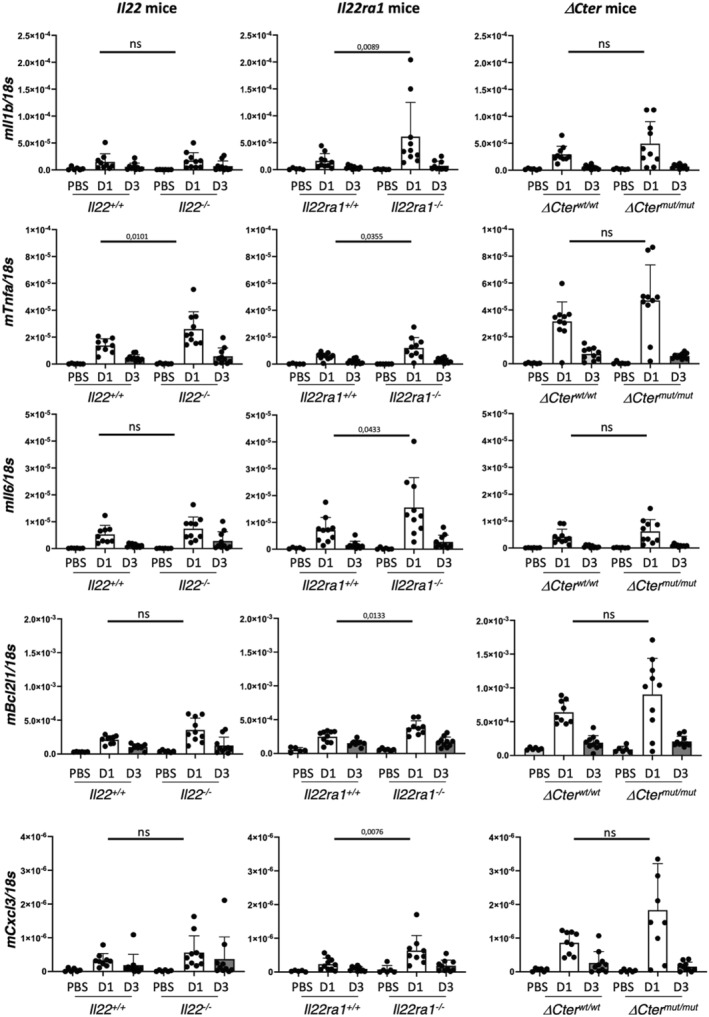
Expression of inflammatory markers after acute pancreatitis induction in *Il22* ^*−/−*^, *Il22ra1* ^*−/−*^ and Δ*Cter* ^*mut/mut*^ mice. RT‐qPCR analysis in healthy (PBS) or caerulein‐injected pancreas (D1 or D3). RNA was extracted and gene expression of *Il1b*, *Tnfa*, *Il6*, *Bcl2l1 and Cxcl3* was analyzed and normalized on *18s* housekeeping gene. D1 = 1 day after caerulein injection. D3 = 3 days after caerulein injection. Data are shown as mean ± SEM and are a pool of 2 (or 3 for *Il22ra* mice) independent experiments with 3–5 mice/group. Statistical analysis between WT and mutant mice treated with caerulein at the same time‐point: Mann–Whitney (ns = not significant).

In addition, the expression of inflammatory markers, *Il1b*, *Tnfa*, and *Il6*, was assessed in the pancreas (Figure 4). Notably, significant induction of these genes was observed only at one day post injury, with gene expression levels returning close to baseline by day 3. In *Il22ra1* ^*−/−*^ mice, the expression of all 3 inflammatory markers was significantly increased compared with control mice, at day one. In *Il22* ^*−/−*^ mice, only the expression of *Tnfa* was significantly increased relative to WT group. No significant differences were detected in the Δ*Cter* mice. Pancreatic *Bcl2l1* and *Cxcl3* expression was also assessed by RT‐qPCR following induction of acute pancreatitis (Figure 4). No significant differences were observed between *Il22* ^*+/+*^ and *Il22* ^*−/−*^ mice at day 1 of caerulein treatment. In contrast, *Il22ra1* ^*−/−*^ mice showed significantly higher pancreatic *Bcl2l1* and *Cxcl3* expression than *Il22ra1* ^*+/+*^ littermates at day 1, consistent with the more severe pancreatic injury observed in these animals. Of note, *Bcl2l1* has previously been described as a downstream target of IL‐22/STAT3 signaling in pancreatic acinar cells,^19^ whereas *Cxcl3* is more likely to reflect an enhanced inflammatory response associated with pancreatitis severity. No significant difference in *Bcl2l1* or *Cxcl3* expression was detected between Δ*Cter* ^*wt/wt*^ and Δ*Cter* ^*mut/mut*^ mice at the same time point, again consistent with the low pancreatic damage severity observed for these mice.

### 3.3 The ADM process relies on both IL‐22 and its receptor, whereas the C‐terminal region of IL‐22R does not impact regeneration

**FIGURE 5:**
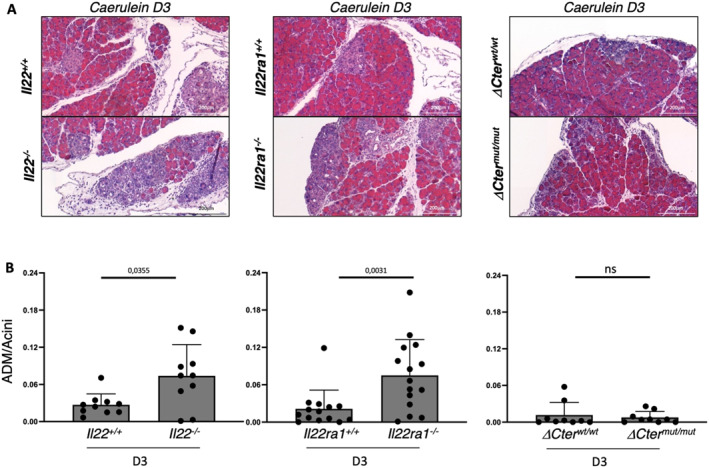
Acinar‐to‐ductal metaplasia is dependent on IL‐22 and IL‐22R expression. (A) H&E staining of pancreas sections of mice treated with caerulein 3 days after initiation of pancreatitis. Magnification x20, scale bar = 200 μm. One representative picture is shown for each group (*n* = 3–5 mice/group). Quantification of ADM based on HE staining (B) was performed using the Halo software. D3 = 3 days after caerulein injection. Data are shown as mean ± SEM and are a pool of 2 (or 3 for *Il22ra* mice) independent experiments with 3‐5 mice/group. Statistical analysis between WT and mutant mice treated with caerulein at the same time‐point: Mann–Whitney (ns = not significant).

At 3 days post injury, corresponding to the regenerative phase during which the pancreas reacquires an acinar phenotype, pancreatic weights returned to baseline levels (Figure 2C). To further evaluate the extent of the acinar‐to‐ductal metaplasia (ADM) process, pancreatic sections were stained with Hemalun and Eosin (Figure 5A). ADM regions can be distinguished from healthy pancreatic tissue by their more basophilic appearance and the presence of duct‐like structure as observed in HE staining. Immunostaining for CK19, a ductal marker, further confirmed the identification of these ADM regions (Supporting Information S1 Figure S3). To quantify ADM, Halo software was used to calculate the ratio of ADM areas to total acinar tissue based on H&E‐stained sections (Figure 5B). This ratio is significantly increased in both *Il22* ^*−/−*^ and *Il22ra1* ^*−/−*^ mice compared with their respective controls. In contrast, no significant differences were observed for Δ*Cter* mice.

Altogether, these results indicate that the absence of the alternative STAT3 pathway, achieved through deletion of C‐terminal region, does not prevent the protective effects mediated by IL‐22Rα signaling during caerulein‐induced acute pancreatitis. The canonical, tyrosine‐dependent pathway appears to be sufficient to confer protection. The lack of involvement of the C‐terminal region may reflect the relatively low level of STAT3 activation required for IL‐22Rα‐mediated protective effects.

## 4 DISCUSSION

Our study confirms the protective role of IL‐22 in pancreatic inflammation, in line with previous reports.^27^, ^28^ In addition, we identify IL‐22Rα1 as a critical regulator of pancreatic injury, whose protective function extends beyond IL‐22 itself. Indeed, IL‐22Rα1 deficiency results in more severe pancreatitis than IL‐22 deficiency, as evidenced by increased serum amylase levels, exacerbated edema, and enhanced acinar‐to‐ductal metaplasia compared with wild‐type mice. In contrast, although the C‐terminal domain of IL‐22Rα1 supports robust STAT3 activation, its absence does not aggravate caerulein‐induced pancreatitis, indicating that maximal STAT3 signaling is not required for IL‐22Rα1‐mediated pancreatic protection.

It is noteworthy that, although the protective effects of exogenous IL‐22 administration are well established, the consequences of IL‐22 deficiency have remained controversial. One study reported exacerbated disease in the absence of IL‐22, whereas another found no significant effect and further suggested that IL‐22 was not produced in their experimental model.^19^ In our study, IL‐22‐deficient mice developed more severe caerulein‐induced pancreatitis, thereby supporting the findings of Huan et al.^18^ and reinforcing the protective role of IL‐22 inferred from exogenous administration studies. One possible explanation for these discrepancies is the differences in experimental design. Notably, our study relied on littermate‐controlled animals, thereby minimizing variations in genetic background and microbiota composition, whereas such controls were not reported previously. Given the well‐established influence of the microbiota on immune responses and pancreatitis severity, these factors may have masked or modulated the impact of IL‐22 deficiency in earlier studies.

Our data further indicate that the alternative signaling pathway contributes to STAT3 activation in the pancreas, consistent with observations in the skin and liver, but not in the intestine.^13^ This suggests that the contribution of this pathway is tissue‐specific. Importantly, the function of IL‐22 in inflammatory conditions appears to be highly context‐dependent. In the setting of acute pancreatitis, where IL‐22 exerts protective effects,^19^ disruption of the alternative pathway does not impair this protective activity.

Several nonmutually exclusive mechanisms may account for this observation. First, the high expression of IL‐22Rα1 in the pancreas^29^ may allow sufficient STAT3 activation through the canonical, tyrosine‐dependent pathway, despite the loss of the C‐terminal region. In this context, receptor abundance may compensate for the absence of alternative signaling motifs, ensuring a level of STAT3 activation adequate to mediate protection. Second, the protective effects of IL‐22 in this model may not rely primarily on STAT3. Other downstream pathways activated by IL‐22Rα1, and unaffected by C‐terminal truncation, may play a dominant role. In particular, pathways such as STAT5 or AKT/mTOR—both implicated in pancreatic protection and the regulation of acute pancreatitis—may contribute to the observed phenotype.^30^, ^31^ Notably, STAT5 activation depends on receptor tyrosine residues, particularly tyrosine 326 of IL‐22Rα1^13^ (and unpublished data), and would therefore remain intact upon deletion of the C‐terminal domain. As this region primarily contributes to STAT3 signaling, its removal would be expected to have limited impact on these parallel pathways.

Taken together, these findings suggest that either residual, tyrosine‐dependent STAT3 activation is sufficient to mediate IL‐22‐driven protection, or that STAT3‐independent pathways play a major role in this process. An interesting translational implication of our study is that selective inhibition of the C‐terminal region of IL‐22 may not interfere with its protective role in acute pancreatitis. Although this domain contributes to STAT3 activation, its deletion did not exacerbate pancreatic injury in our model, suggesting that it is not essential for IL‐22‐mediated protection in this setting. In contrast, we previously showed that the C‐terminal region promotes disease development in Imiquimod‐induced psoriasis, as ΔCter^mut/mut^ mice were partially protected from skin inflammation.^13^ These findings raise the possibility that therapies selectively targeting the C‐terminal region of IL‐22R could reduce its pathogenic activity in psoriasis while preserving its beneficial functions in other tissues. Further studies will be required to evaluate this therapeutic concept in additional disease settings.

Finally, we observed that IL‐22Rα1 deficiency leads to more severe pancreatic damage than IL‐22 deficiency alone. This may be explained by the fact that IL‐22Rα1 is not exclusively engaged by IL‐22 but also serves as a receptor subunit for IL‐20 and IL‐24 when heterodimerized with IL‐20Rβ. Consequently, the phenotype observed in *Il22ra1* ^*−/−*^ mice likely reflects the combined loss of signaling mediated by IL‐22 and other IL‐20 family cytokines. Although increased expression of IL‐24 and its receptor have been reported in chronic pancreatitis, the functional role of IL‐20‐related cytokines in pancreatic inflammation remains poorly characterized.^32^ Our findings therefore suggest that these cytokines may contribute to pancreatic tissue protection or repair and warrant further investigation in the context of acute pancreatitis.

Although our conclusions are based on a well‐established experimental model, one limitation of the present study is that all experiments were performed in mouse models, without validation in human pancreatic tissue. However, access to human pancreatic samples from patients with acute pancreatitis is extremely limited, as pancreatic biopsies are not routinely performed because of the substantial procedural risks. As a result, well‐characterized human pancreatic tissue suitable for mechanistic investigations is exceedingly rare. This limitation largely explains why most studies exploring the role of IL‐22 in acute pancreatitis have relied on experimental animal models rather than human pancreatic specimens. Although pancreatic tissue may occasionally be obtained from patients undergoing surgery for chronic pancreatitis, the isolation and long‐term maintenance of viable primary human acinar cells remain technically challenging. Moreover, chronic pancreatitis is characterized by extensive fibrosis, acinar cell loss, and acinar‐to‐ductal metaplasia, substantially reducing the availability of physiologically relevant acinar cells for functional studies. Consequently, direct characterization of IL‐22 signaling in primary human pancreatic acinar cells has not yet been achieved. Future studies integrating clinically relevant human models, including patient‐derived pancreatic organoids or ex vivo tissue systems as they become available, will be important to further validate the translational relevance of our findings.

## AUTHOR CONTRIBUTIONS

All authors confirmed substantial contributions to conception and design of, or acquisition of data or analysis and interpretation of data, drafting the article or revising it critically for important intellectual content and final approval of the version to be published.

## CONFLICT OF INTEREST STATEMENT

The authors declare no conflicts of interest.

## ETHICS STATEMENT

All mouse experiments were conducted in accordance with national and institutional guidelines and were approved by the local ethical committee (LA 1230653‐2023/UCL/MD/08).

## Supporting information

Supporting Information S1

## Data Availability

The data that support the findings of this study are available on request from the corresponding author. The data are not publicly available due to privacy or ethical restrictions.
