# Supplementary material for: IL‐22Rα1 restrains pancreatic injury independently of its C‐terminal STAT3‐amplifying domain
Source: J Cell Commun Signal. 2026 Jul 28;20(3):e70098. doi: 10.1002/ccs3.70098 (PMC13412541; doi:10.1002/ccs3.70098)
Supplement: Supplementary file 1 — Supporting Information S1 [file CCS3-20-e70098-s001.docx]

**Supplementary Figures**

**
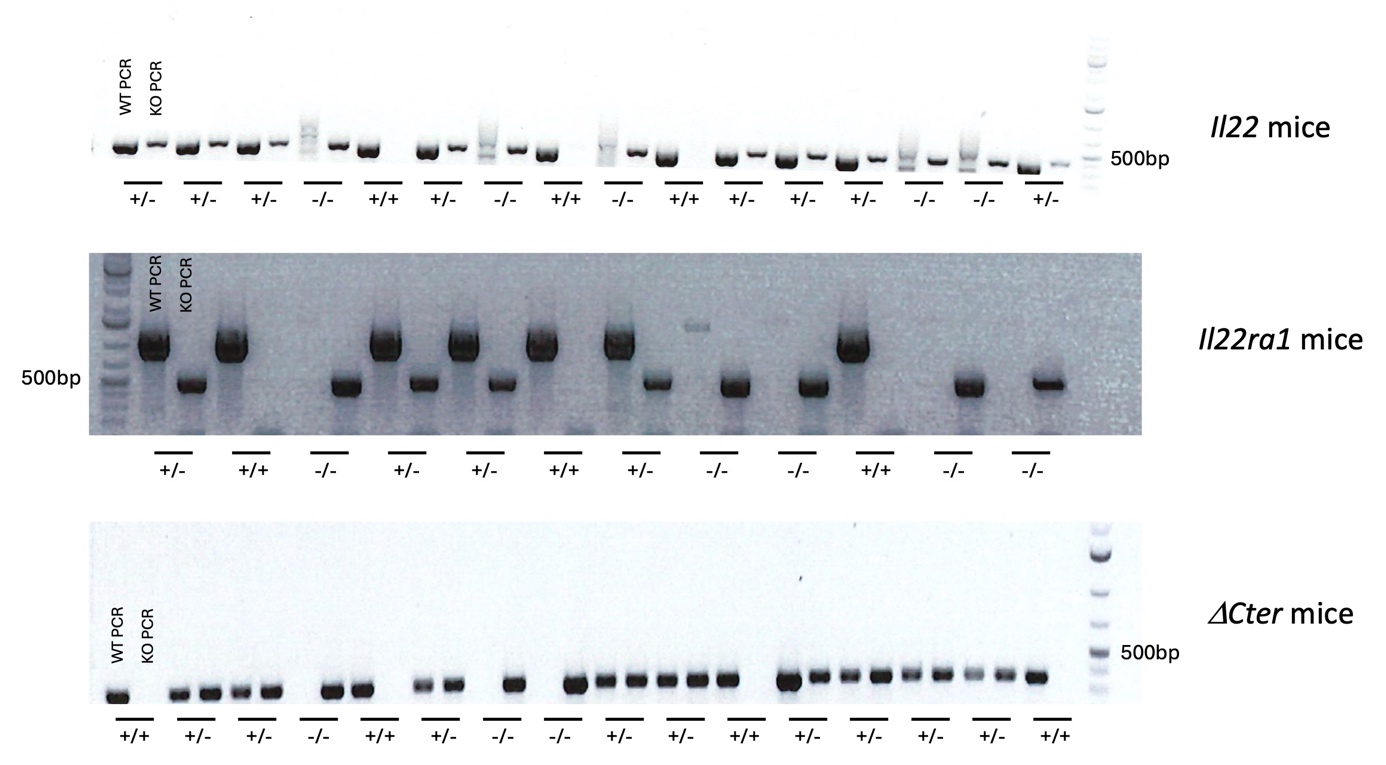
**

**Suppl. Figure 1**

**Genotyping of littermate mice.**

Representative PCR genotyping of littermate mice using allele-specific primers for the WT and KO alleles. Genomic DNA from each mouse was subjected to two separate PCR reactions. WT mice showed amplification exclusively with the WT primer set, whereas KO mice showed amplification exclusively with the KO primer set. Full protocol is displayed in the Methods sections.


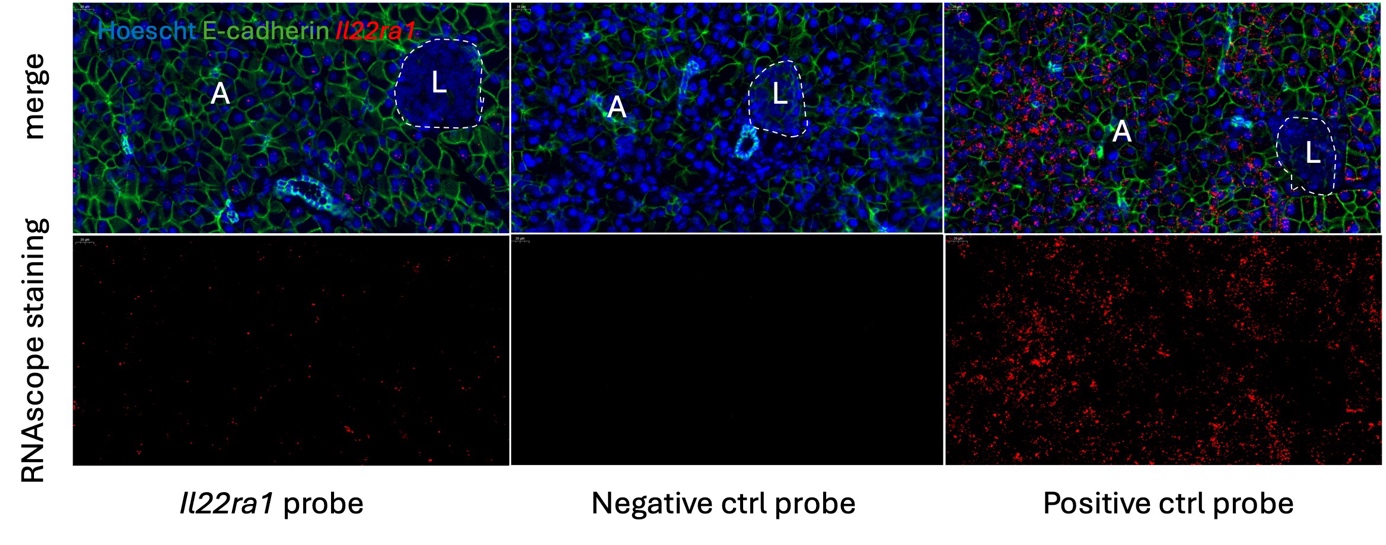


**Suppl. Figure 2**

RNA-scope images for Il22ra1 staining on pancreas sections of WT mice. **L** represents Langerhans islets and **A** the acinar parenchyma. Staining of nuclei with Hoechst (blue), Il22ra1(red) and E-Cadherin (green) Magnification x40, scale bar = 20μm.


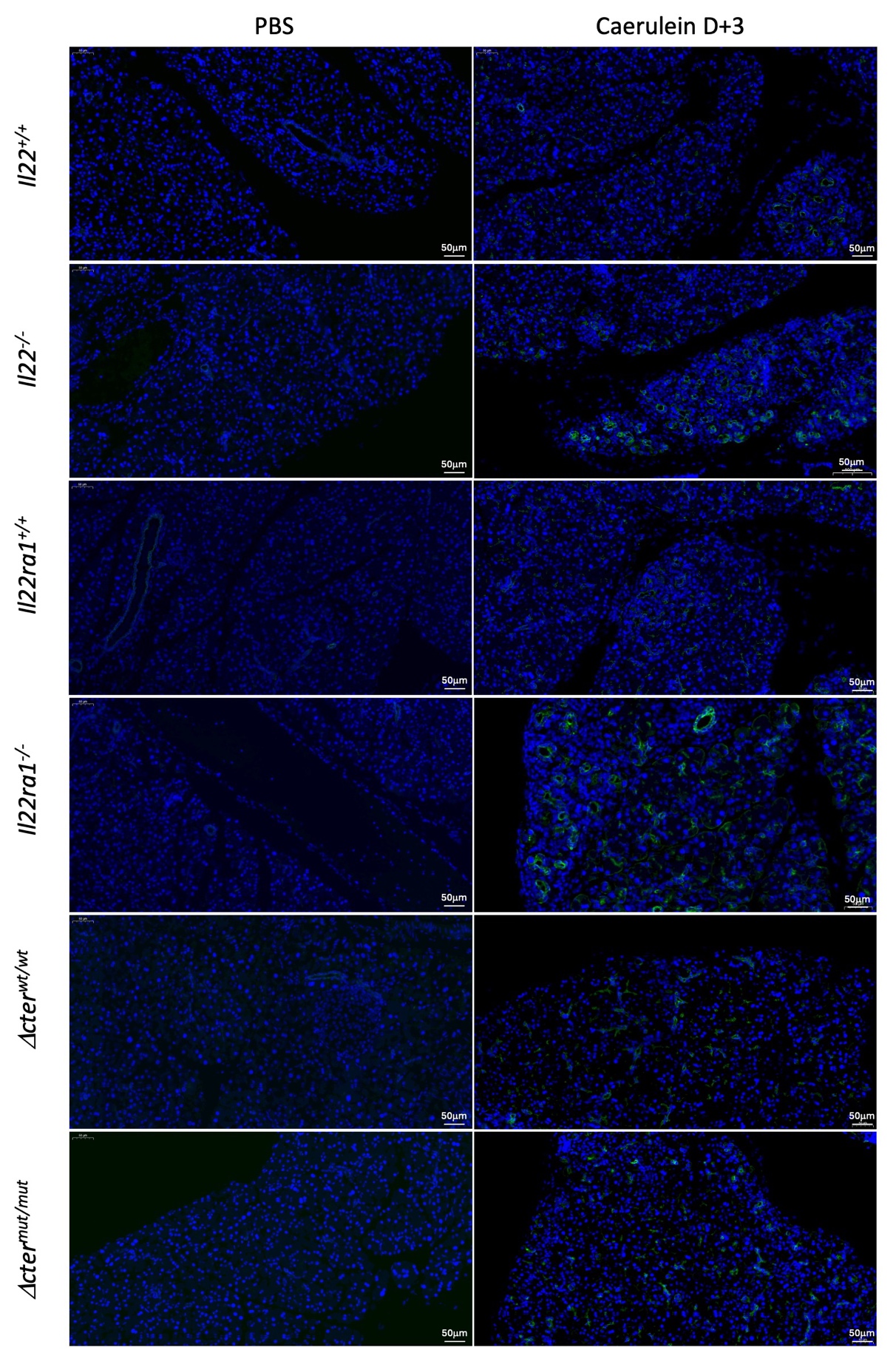


**Suppl. Figure 3**

CK19 staining of pancreas sections of mice treated with caerulein 3 days after initiation of pancreatitis. Magnification x20, scale bar = 50μm. One representative picture is shown for each group (n= 3-5 mice/group). Nuclei are stained in blue and CK19 in green.
